# 4-Iodobenzonitrile as Effective Solid Additive for High-Efficiency Polymer Solar Cells

**DOI:** 10.3390/polym17101386

**Published:** 2025-05-18

**Authors:** Jiayu Li, Chuanchen Cai, Yuechen Li, Changbiao Ma, Sergio Gámez-Valenzuela, Yixiao Liu, Jianfeng Li, Xiaochen Wang, Yongfang Li

**Affiliations:** 1 School of Materials Science and Engineering, Shaanxi Normal University, Xi’an 710119, China; 2 Beijing National Laboratory for Molecular Sciences, CAS Key Laboratory of Organic Solids, Institute of Chemistry, Chinese Academy of Sciences, Beijing 100190, China; 3 Shenzhen Key Laboratory of Printed Electronics, Department of Materials Science and Engineering, Southern University of Science and Technology, Shenzhen 518055, China

**Keywords:** polymer solar cells, solid additives, morphology control

## Abstract

Solid additive engineering is a well-established and effective strategy for enhancing active layer morphology in polymer solar cells (PSCs), thereby improving their power conversion efficiency (PCE). However, the availability of effective solid additive molecules remains limited, especially those combining simple structural units with a large dipole moment to promote strong interactions with active materials. In this study, we introduce 4-iodobenzonitrile (IBZN), a commercially available, low-cost, and structurally simple molecule with a high dipole moment (3.33 debye), as a solid additive for PSCs. Theoretical calculations, ultraviolet–visible (UV-Vis) spectroscopy experiments, and a morphology analysis demonstrate that IBZN forms strong interactions with L8-BO, subsequently enhancing the packing mode and crystallization. The incorporation of IBZN into PM6:L8-BO-based PSCs resulted in an increased fill factor (FF) of 79.54% and a boosted PCE from 17.49% to 18.77%. Furthermore, IBZN has also demonstrated outstanding regulatory effects in systems based on other Y-series acceptors, such as Y6 and BTP-ec9. This study not only introduces a structurally simple solid additive molecule characterized by a large dipole moment but also offers valuable insights for the subsequent development of novel solid additives aimed at enhancing the morphology and efficiency of PSCs.

## 1. Introduction

Polymer solar cells (PSCs) have attracted considerable interest owing to their low cost, solution processability, light weight, and mechanical flexibility [1,2,3,4,5,6,7,8]. Recent advancements in Y-series derivative materials and optimized device fabrication techniques have enabled PSC efficiencies to exceed 20% [9,10,11,12,13,14,15,16,17,18]. Typically, the performance of PSCs is significantly limited by the film morphology of the active layer. Achieving optimal phase separation and a well-defined donor–acceptor nanoscale interpenetrating network at the microscale is crucial for efficient exciton dissociation and subsequent charge transport [19,20,21,22]. Device processing methods such as thermal annealing [23], solvent vapor annealing [24], and additive engineering [25] are alternative strategies for controlling active layer morphology. Among these, additive engineering, a facile and effective auxiliary technique, plays a significant regulatory role in active layer film formation.

Traditional high-boiling-point liquid additives, including 1,8-diiodooctane (DIO) [26] and 1-chloronaphthalene (1-CN) [27,28], were extensively used in fullerene-based systems. By selectively solubilizing the active layer components and retarding solvent evaporation, these additives balanced donor and acceptor crystallization, thereby improving the morphology and optimizing charge separation and transport. However, the gradual volatilization of residual additives, which are challenging to completely remove, can compromise the long-term device stability [29,30]. Furthermore, their high volume sensitivity can readily lead to excessive donor–acceptor phase separation scales, deteriorating the film morphology and device performance. Solid additives [31,32], developed more recently, can induce alterations in the arrangement and orientation of active layer molecules, adjust stacking distances, and enhance crystallinity through various non-covalent interactions, thereby promoting charge separation and extract. These solid additives are readily removed from the film, enhancing device reproducibility. Commonly employed solid additives typically exhibit the following characteristics: (1) low cost and simple synthesis for large-scale application; (2) the presence of polar groups such as halogen atoms or cyano groups enables the additive molecules to maintain a certain dipole moment [33], where appropriate dipole interactions can modulate the orientation and arrangement of active component molecules; (3) inherent high crystallinity [34], acting as nucleation sites for active components during film formation, thus inducing improved molecular crystallization behavior.

Recently, substantial efforts have been dedicated to the development of volatile solid additives. For example, Yuan et al. [35] developed several volatile solid additives based on electron-deficient imide structural units. N-(p-chlorophenyl)phthalimide (pClPA), possessing a maximum dipole moment of 4.79 debye, altered the nucleation sequence of the acceptor, significantly improving the film crystallinity and achieving an excellent PCE of 19.04% in the D18:L8-BO system. Gong et al. [36] reported a synergistic liquid–solid dual-additive strategy, where 2,5-dibromo-3,4-thiophenedinitrile (DHT), with a large dipole moment of 5.91 debye, exhibited strong interactions with the acceptor, significantly enhancing electron mobility and assisting in the performance of the PM6:Y6 system. However, the synthesis of such high-dipole moment molecules often involves complex procedures, hindering their widespread application. Some structurally simple additives, such as those based on benzene units, have also been extensively investigated. Hou et al. [37] reported the utilization of a solid additive, 1,3,5-trimethoxybenzene (TMB), aimed at optimizing film morphology and minimizing energy disorder, resulting in a PCE of 18.61% in a device based on PM6:BTP-ec9. In a similar vein, Kan et al. [38] explored several volatile solid additives which were demonstrated to modulate molecular aggregation and crystallinity of the active layer by engaging in halogen bonding with neighboring Y6 molecules. A high PCE of 17.9% has been achieved based on the PM6:Y6 system. However, while these halogen-substituted benzene unit-based additives have simple structures, they typically possess relatively small molecular dipole moments. Molecules with higher dipole moments can generally exhibit stronger interactions with active component molecules, further modulating molecular orientation [39]. Actually, the current availability of effective solid additive molecules remains limited, particularly those integrating simple structural units and large dipole moments to significantly enhance the PCE of PSCs.

In this work, we selected a low-cost, commercially available, and structurally simple molecule, 4-Iodobenzonitrile (IBZN), which features cyano and iodine atoms para-substituted on a benzene unit. The highly electron-withdrawing cyano group and the less electron-negative iodine atom endow IBZN with a relatively large dipole moment (3.33 debye). As a facile and effective regulation tool, theoretical calculations and ultraviolet–visible spectroscopy experimental results demonstrate that IBZN can generate strong interactions with L8-BO molecules, inducing molecular aggregation and enhancing crystalline orientation. Simultaneously, IBZN can promote the miscibility between active layer materials, facilitating the ordered stacking of active layer molecules. Benefiting from the more uniform and smoother film and the controlled phase separation scale, exciton dissociation and charge transport performance are improved. Ultimately, compared to the control device (PCE of 17.49%), the IBZN-optimized device based on the PM6:L8-BO system exhibits an excellent fill factor (79.54%) and an enhanced PCE of 18.77%. Moreover, IBZN demonstrates good applicability in established Y-series acceptors such as Y6 and BTP-ec9. The results demonstrate that IBZN, characterized by a low cost and high dipole moment, effectively enhances intermolecular interactions with the acceptors, optimizes active layer crystallization and improves the film morphology, consequently leading to a significant enhancement in the PCE of PSCs. These findings offer valuable insights for the future development of cost-effective, high-performance additives in PSCs.

## 2. Materials and Methods

The donor polymer PM6, acceptor molecules L8-BO, Y6, and BTP-ec9, along with the electron transport layer (ETL) material PDINN, were procured from Nanjing Zhiyan Biotechnology Co., Ltd. Nanjing, China. The hole transport layer (HTL) material PEDOT:PSS (Poly (3,4-ethylenedioxythiophene)-poly (styrenesulfonate)) was obtained from Xi’an Yuri Solar Co., Ltd. Xi’an, China. The additive 4-Iodobenzonitrile (IBZN), deuterated chloroform (CDCl_3_, 99.6 atom % D, stabilized with silver), and methanol (HPLC grade) were purchased from TCI Development Co., Ltd. Shanghai, China. All materials were used as received without further purification.

PSCs were fabricated with an architecture of ITO (150 nm)/PEDOT:PSS (40 nm)/Active layer (120 nm)/PDINN (10 nm)/Ag (110 nm). Indium tin oxide (ITO)-coated glass substrates were initially cleaned by sequential ultrasonication in deionized water, detergent, acetone, and isopropyl alcohol, followed by drying in an oven at 120 °C for 4 h. The hole transport layer was prepared by spin-coating the PEDOT:PSS onto the cleaned ITO substrates at 5000 rpm for 20 s, followed by drying at 150 °C for 15 min. Active layer solutions were prepared by dissolving the donor polymer (PM6) and acceptor molecules (L8-BO, Y6, or BTP-ec9) in chloroform (CF) at a donor-to-acceptor weight ratio of 1:1.2. The concentrations of the donor polymers were 7 mg/mL for PM6:L8-BO and PM6:BTP-ec9 blends, and 7.5 mg/mL for the PM6:Y6 blend. These solutions were stirred at 45 °C for 2 h. The additive IBZN was incorporated into the respective active layer solutions at varying weight percentages relative to the acceptors (40 wt%, 70 wt%, 100 wt%, and 130 wt%). Subsequently, the active layer solutions were spin-coated onto the PEDOT:PSS layer at spin speeds ranging from 2500 to 3000 rpm and thermally annealed at specific temperatures (90 °C, 100 °C, and 110 °C) for 10 min. The electron transport layer was deposited by spin-coating a PDINN solution (1 mg/mL in methanol) onto the active layer at 3000 rpm. Finally, a 100 nm thick silver (Ag) top electrode was deposited by thermal evaporation under a vacuum of 2.0 × 10^−4^ Pa. The active area of each device was 0.045 cm^2^, defined by a shadow mask.

## 3. Results and Discussion

**Figure 1:**
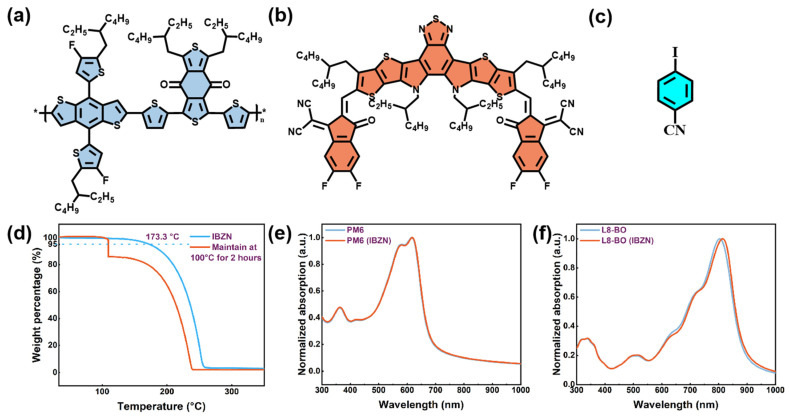
The molecular structure of (**a**) PM6, (**b**) L8-BO, (**c**) IBZN. (**d**) The TGA curve of IBZN. The UV-Vis absorption spectra of (**e**) PM6 and (**f**) L8-BO films before and after the incorporation of 70 wt% IBZN.

The molecular structures of PM6, L8-BO, and IBZN are depicted in Figure 1. IBZN is derived from the substitution of a cyano group and an iodine atom at the para position of the benzene unit. The volatility of this additive was initially investigated by a thermogravimetric analysis (TGA), presented in Figure 1d, revealing a 5% weight loss of IBZN at 173.3 °C. Moreover, the sustained heating of IBZN powder at about 100 °C for two hours resulted in a substantial weight reduction. To ascertain its removability from the PM6: L8-BO blend film, Fourier transform infrared (FTIR) spectroscopy was conducted. The infrared absorption spectrum of a pure IBZN pressed pellet is shown in Figure S1. Changes in the absorption peaks at 2227.8, 1474.8, 818.8, and 540 cm^−1^ were monitored to determine the presence or absence of IBZN in the blend film. Notably, these characteristic signals attributed to IBZN were no longer detectable after the blend film underwent annealing at 100 °C for 10 min. Furthermore, the IBZN film was deposited on a silicon substrate (Figure S2). Upon heating to 100 °C, the white powdery crystalline IBZN film sublimed, and the substrate surface recovered its smooth and lustrous state. These findings confirm the favorable volatility and removability of IBZN, indicating that it does not directly engage in the photoelectric conversion processes within the device active layer.

To analyze the effects of IBZN on the packing and aggregation of the donor and acceptor molecules, UV-Vis spectroscopy measurements were performed on several films, as illustrated in Figure 1e,f. The inclusion of IBZN resulted in a minimal impact on the absorption spectrum of the PM6 film, manifesting only as a slight enhancement in the 0-0/0-1 vibronic peak intensity ratio and a subtle redshift. In contrast, the L8-BO film exhibited more significant spectral alterations upon additive incorporation, with the absorption peak undergoing a redshift from 802 nm to 814 nm. This observation implies that IBZN modifies the molecular packing of the acceptor, promoting a higher degree of aggregation in its solid state [40]. The absorption coefficient curves of the blend films are presented in Figure S3. The additive-processed film demonstrates an elevated absorption coefficient across the entire spectral range. Concurrently, the slightly red-shifted absorption in the acceptor region also leads to a spectral broadening, suggesting an enhanced potential for the active layer to harvest solar photons [41], which is favorable for improving the short-circuit current density.

**Figure 2:**
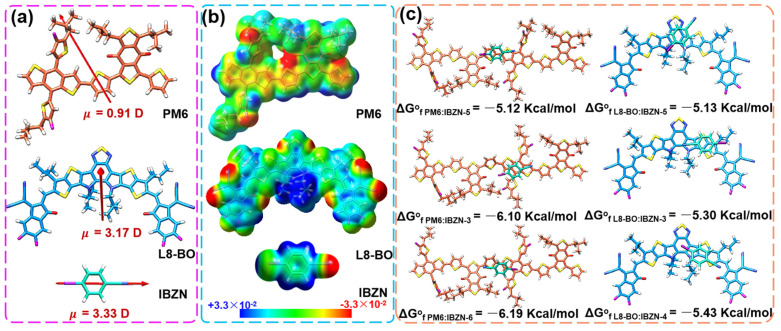
(**a**) The calculated dipole moment of PM6, L8-BO, IBZN, and (**b**) ESP distribution on the surface of PM6, L8-BO, and IBZN; (**c**) top views and Gibbs free energies of formation for the molecular complexes of PM6:IBZN and L8-BO:IBZN.

A theoretical analysis was conducted to elucidate the potential interactions between IBZN and the donor–acceptor materials. The molecular geometries for a dimeric model of PM6 polymer, L8-BO molecular acceptor and the IBZN additive were optimized at the density functional theory (DFT) level using the GAUSSIAN16 program. The hybrid generalized gradient approximation (GGA) functional B3LYP [42,43] was used together with the LANL2DZ basis set [44,45]. These calculations include an explicit D3 correction for dispersion [46]. The global minimum geometries of the donor polymer PM6 dimer, the acceptor molecule L8-BO, and the additive molecule IBZN were initially optimized using density functional theory (DFT) at the B3LYP-D3/LANL2DZ level, as shown in Figure S4 and Table S1. Meanwhile, the molecular dipole moments were calculated, as depicted in Figure 2a. The dipole moments of PM6 and L8-BO were found to be 0.91 and 3.17 debye, respectively, whereas for IBZN, the asymmetric para-substitution on the benzene unit, coupled with the strong electron-withdrawing nature of the cyano group, induces a significant displacement of the positive and negative charge centers, resulting in a substantial dipole moment of 3.33 debye. The higher dipole moment of the IBZN molecule may induce strong intermolecular interactions with the active layer molecules. Subsequently, we calculated the surface electrostatic potential (ESP) distributions of the three molecules, as illustrated in the accompanying Figure 2b. The rigid conjugated backbone of PM6 predominantly exhibits a negative ESP distribution, while L8-BO displays a predominantly positive ESP distribution, with negative ESP regions localized around the nitrogen, fluorine, and oxygen atoms. Concurrently, the negative ESP distribution of the IBZN molecule is primarily concentrated on the nitrogen atom of the cyano group. Based on the principle of the opposite polar electrostatic attraction, IBZN and L8-BO are anticipated to exhibit stronger intermolecular interactions [47]. To gain insight into the complexation behavior of the IBZN additive with the PM6 donor and L8-BO acceptor, and to evaluate the strength of their intermolecular interactions, the most energetically favorable supramolecular arrangements were evaluated. Six plausible molecular arrangements were considered for each system, evaluating the interaction of the IBZN additive with different moieties of the PM6 polymer and L8-BO small molecule, in both the parallel and antiparallel orientations (Figure S5). As a first view, the IBZN additive exhibited moderate intermolecular interactions with the conjugated backbones of the PM6 donor and L8-BO acceptor, giving rise to diverse complexes through backbone interactions. Specifically, regarding the contact sites in L8-BO-based complexes, the IBZN additive exhibits a clear preference for interacting in the vicinity of the dithienopyrrole (DTP) unit rather than with the benzothiadiazolopyrrole (BTD) unit or the external moieties, as evidenced by the 2–5 Kcal/mol more negative Gibbs free energy of formation (Figure 2c) and higher interaction energies (Table S2). On the other hand, the additive is inclined to adhere to the benzodithiophene moiety of the polymer donor PM6. In this case, Gibbs free energy differences of 4 kcal/mol were calculated for these complexes when compared to others in which IBZN was interacting with the benzodithiophene-dione unit or pendant thiophene rings. Therefore, the presence of intermolecular interactions between IBZN and the active materials is evident.

**Figure 3:**
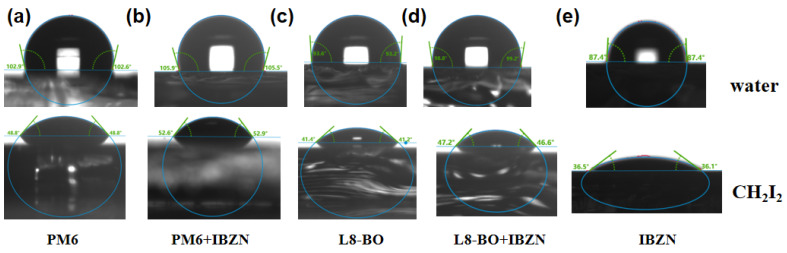
Water and diiodomethane contact angle measurements on the (**a**) PM6 film, (**b**) PM6+IBZN film, (**c**) L8-BO film, (**d**) L8-BO+IBZN film, and (**e**) IBZN film.

Contact angle measurements were performed to further assess the miscibility of the additive with the donor and acceptor materials. Figure 3 illustrates the contact angles of water and diiodomethane on various films. The surface energies (γ) of PM6, L8-BO, and IBZN were determined to be 34.98, 39.7, and 42.8 mN m^−1^, respectively. Subsequently, the Flory–Huggins interaction parameter (χ) was calculated, where a smaller χ value signifies greater miscibility [48]. The calculated χ values for the interaction between IBZN and PM6, IBZN, and L8-BO, are 0.394 and 0.058, respectively. This suggests a higher degree of miscibility between the additive and the acceptor. Consequently, the presence of IBZN is likely to preferentially modulate the assembly behavior of the acceptor molecules, as will be evidenced by the morphology analysis detailed below. We also investigated the effect of IBZN on the miscibility between the donor and acceptor. Upon incorporating IBZN into L8-BO, a substantial change in the film’s surface energy was observed, decreasing from 39.7 mN m^−1^ in the control sample to 35.95 mN m^−1^. As a result, the χ value between PM6 and L8-BO decreased from 0.149 to 0.076, indicating enhanced donor–acceptor miscibility [49]. This improved miscibility may facilitate the evolution of the film morphology towards a more favorable configuration during processing. Detailed calculations are provided in Tables S3 and S4.

**Figure 4:**
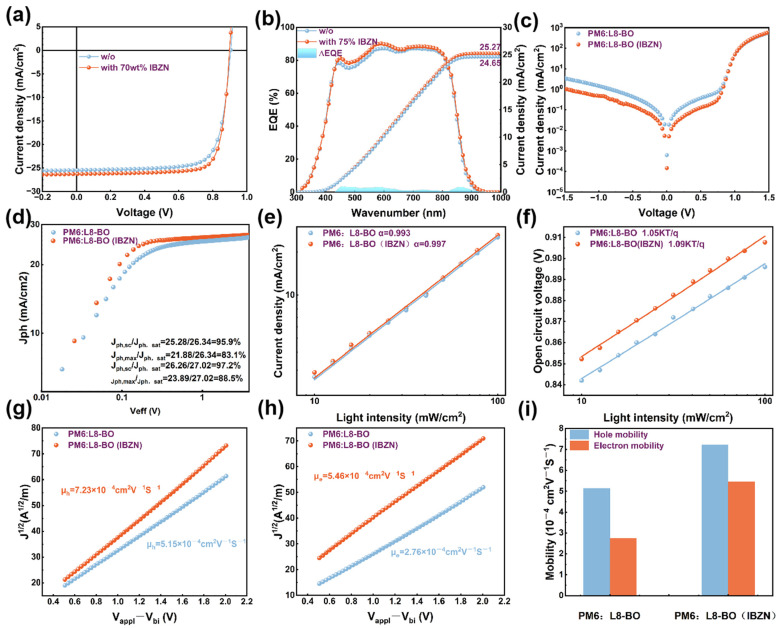
(**a**) *J*-*V* characteristics and (**b**) EQE measurements. (**c**) Dark *J*-*V* characteristics. (**d**) Exciton dissociation efficiency measurements (*J*_ph_-*V*_eff_ curve) of devices. (**e**) Light intensity dependence of (**e**) *J*_SC_ and (**f**) *V*_OC_, *J*^1/2^-*V* curves obtained via the space-charge-limited current (SCLC) method, including (**g**) hole mobility and (**h**) electron mobility based on the PM6: L8-BO blend. (**i**) Mobility comparison plot for the two devices.

To quantitatively evaluate the impact of the IBZN additive on device performance, devices with the structure ITO/PEDOT: PSS/active layer/PDINN/Ag were fabricated. The key photovoltaic parameters, including open-circuit voltage (*V*_OC_), short-circuit current density (*J*_SC_), and FF, were extracted from current density–voltage (*J-V*) curves measured under standard AM1.5G solar illumination, as presented in Figure 4a. The control device exhibited a PCE of 17.49%, with a *V*_OC_ of 0.905 V, a *J*_SC_ of 25.46 mA cm^−2^, and an FF of 75.87%. Following optimization with the IBZN additive under the determined optimal conditions, a significant enhancement in device performance was observed. While the *V*_OC_ experienced a marginal decrease compared to the control device, both the *J*_SC_ and FF were notably improved to 26.28 mA cm^−2^ and 79.54%, respectively, leading to a significantly increased PCE of 18.77%. The corresponding photovoltaic performance parameters are summarized in Table 1. Figure 4b displays the external quantum efficiency (EQE) spectra of both the control and optimized devices. In comparison to the control device, the IBZN additive-optimized device demonstrates an increased spectral response within the 450–800 nm wavelength range. Furthermore, the response is elevated in the 800–950 nm range due to the slight redshift of the active layer absorption band edge, which is consistent with the UV-Vis spectroscopy changes observed in the blend films. The integrated current densities derived from the EQE measurements for the control and optimized devices are 24.65 and 25.27 mA cm^−2^, respectively, exhibiting a deviation of less than 4% from the *J*_SC_ values obtained from the *J-V* characteristics under AM1.5G illumination. The dark current density–voltage (*J-V*) characteristics of the fabricated devices were subsequently evaluated (Figure 4c); the IBZN-optimized device demonstrated a lower current density under reverse bias, and this reduction in reverse leakage current correlated with the improved photovoltaic performance. Detailed optimization procedures, including the weight ratio of the additive and the active layer annealing temperature, are provided in Figure S6 and Table 2 and Table 3. To further expand our understanding, we also investigated the influence of other halogen-substituted analog molecules on the photovoltaic performance of PM6:L8-BO-based devices, including 4-fluorobenzonitrile (FBZN), 4-chlorobenzonitrile (CBZN), and 4-bromobenzonitrile (BBZN). The structures of these three molecules are illustrated in Figure S7, while the optimized device performance parameters are presented in Table S5, with the corresponding *J-V* curves shown in Figure S8.

To further elucidate the exciton dissociation and charge collection processes, the photocurrent density (*J*_ph_) as a function of effective voltage (*V*_eff_) was analyzed [50]. Here, *J*_ph_ is defined as the difference between the current density under illumination (*J*_L_) and in the dark (*J*_D_), and *V*_eff_ is defined as the difference between the *V*_0_ (*V*_0_ represents the voltage where the *J*_L_ is equivalent to the *J*_D_) and the applied bias voltage (*V*_apply_). The exciton dissociation efficiency (*η*_diss_) and charge collection efficiency (*η*_coll_) were determined from the ratios *J*_ph_/*J*_sat_ (photocurrent density under short-circuit conditions normalized by the saturation photocurrent density) and *J*_max_/*J*_sat_ (photocurrent density at the maximum power point normalized by the saturation photocurrent density), respectively. As illustrated in Figure 4d, the IBZN-modified device achieved photocurrent saturation at a lower effective voltage, yielding calculated *η*_diss_ and *η*_coll_ values of 97.2% and 88.5%, respectively. In contrast, the control device exhibited *η*_diss_ and *η*_coll_ values of 95.9% and 83.1%, respectively. Detailed calculations are provided in Table S6. The enhanced exciton dissociation and charge collection efficiencies contributed to the higher *J*_SC_ and FF observed in the modified device.

The charge recombination behavior within the devices was further investigated by analyzing the dependence of *V*_OC_ and *J*_SC_ on light intensity (*P*_light_). The relationship between *V*_OC_ and *P*_light_ follows a power law, *J*_SC_ ∝ *P*_light_ ^α^, where a value of α closer to 1 indicates reduced bimolecular recombination. The relationship between *V*_OC_ and *P*_light_ is given by *V*_OC_ ∝ (nkT/q)ln(*P*_light_), where k is the Boltzmann constant, T is the absolute temperature, and q is the elementary charge. A factor n close to 1 suggests that bimolecular recombination dominates, while a value close to 2 indicates prevalent trap-assisted recombination. As shown in Figure 4e, the control device exhibited an α value of 0.993, and the IBZN-modified device showed an α value of 0.997, indicating suppressed bimolecular recombination in both cases. Figure 4f presents the *V*_OC_ versus ln(*P*_light_) plot, the ideality factor n for the IBZN-containing device was determined to be 1.05kT/q, which is lower than the 1.09kT/q observed for the control device, suggesting a reduction in trap-assisted recombination. This improved charge transport performance is consistent with the observed enhancement in device efficiency.

Changes in carrier mobility partially reveal the charge transport capability of the device. Consequently, the hole (*μ*_h_) and electron (*μ*_e_) mobilities of the devices were evaluated using the space-charge-limited current (SCLC) method, with carrier mobilities extracted by fitting the *J*^1/2^-*V* plots [51], as shown in Figure 4g–i. The control device exhibited *μ*_h_ and *μ*_e_ values of 5.15 × 10^−4^ cm^2^ V^−1^ s^−1^ and 2.76 × 10^−^⁴ cm^2^ V^−1^ s^−1^, respectively, resulting in a *μ*_h_/*μ*_e_ ratio of 1.86. Following additive modification, *μ*_h_ and *μ*_e_ increased to 7.23 × 10^−4^ cm^2^ V^−1^ s^−1^ and 5.46 × 10^−4^ cm^2^ V^−1^ s^−1^, respectively, leading to a more balanced *μ*_h_/*μ*_e_ ratio of 1.32. Detailed calculations are provided in Table S7. Compared to the control device, the incorporation of IBZN resulted in a more pronounced enhancement in both *μ*_h_ and *μ*_e_, and more balanced charge carrier transport, mitigating single-carrier accumulation and the associated increase in charge recombination. This improvement may be attributed to the more ordered molecular arrangement and enhanced film crystallinity, which will be discussed in the section describing the film morphology analysis.

As discussed earlier, device performance is strongly correlated with the active layer morphology. Consequently, modifications in the aggregation behavior of active layer constituent molecules, molecular alignment, and crystallinity will have a substantial impact on charge separation and charge carrier transport. The impact of the additive on the morphology of films was initially examined using atomic force microscopy (AFM). As depicted in Figures S9 and S10, the surface morphology of the pristine PM6 film remained largely unaltered upon additive incorporation, with surface roughness (R_q_) values of 1.10 nm and 1.13 nm before and after treatment, respectively. This suggests a limited influence of the additive on the solid-state aggregation of the donor. Conversely, the pristine L8-BO film exhibited a significant change in surface roughness. The control L8-BO film displayed a low R_q_ of 0.52 nm, whereas the inclusion of the additive resulted in a distinct modification of the acceptor’s aggregation behavior, leading to a substantial increase in R_q_ to 3.04 nm. The phase image and 3D height map revealed a notable enlargement of the acceptor phase domain size and a discernible enhancement in crystallinity. To further elucidate the alterations in acceptor molecular packing and crystallinity, grazing-incidence wide-angle X-ray scattering (GIWAXS) measurements were conducted on the acceptor films. Figure S11 presents the 2D-GIWAXS patterns and the corresponding one-dimensional line-cut profiles in the out-of-plane (OOP) and in-plane (IP) directions. The pristine L8-BO film exhibited a prominent (010) π-π stacking signal at a scattering vector (q) of 1.74 Å^−1^ in the OOP direction, corresponding to a π-π stacking distance of 3.61 Å, indicative of a preferential face-on orientation [52]. Following the IBZN treatment, the intensity of the (010) diffraction signal in the OOP direction for the L8-BO film increased, and the diffraction peak shifted to q = 1.75 Å^−1^, corresponding to a reduced π-π stacking distance of 3.59 Å. The crystalline coherence length in the OOP direction increased from 21.18 Å to 26.8 Å. Furthermore, the intensity of the (100) lamellar stacking diffraction signal in the IP direction also increased, and the stacking distance decreased from 13.08Å to 12.61Å. These observations demonstrate the additive induces changes in the L8-BO molecular orientation, resulting in a more compact molecular packing and a significantly enhanced crystalline order, consistent with the preceding discussion.

**Figure 5:**
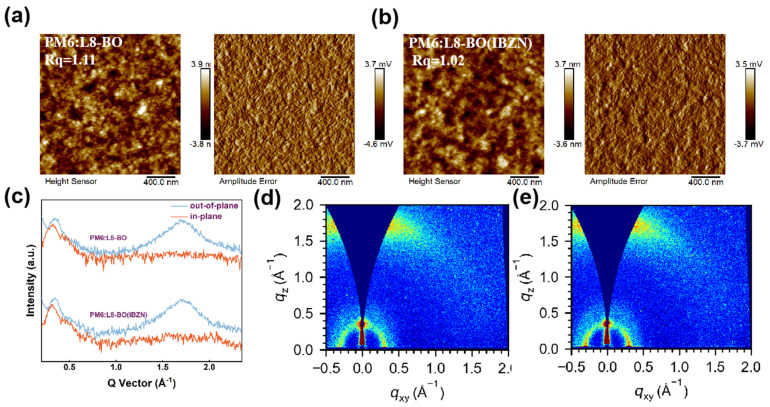
The height map and phase map of AFM (from left to right) of (**a**) the pristine PM6: L8-BO film and (**b**) PM6: L8-BO films containing the additive IBZN. (**c**) The one-dimensional (1D) line cuts along the out-of-plane (OOP) and in-plane (IP) directions for both two PM6: L8-BO films. The 2D-GIWAXS patterns of (**d**) the pristine PM6: L8-BO film and (**e**) PM6: L8-BO film with the incorporated IBZN, respectively.

Considering that the morphology, molecular orientation, and crystallinity changes in the blend film directly influence the charge transfer processes, thereby affecting device performance, further investigation was performed on the blend films. As shown in Figure 5, the pristine PM6:L8-BO blend film exhibited a surface roughness of 1.11 nm. After additive treatment, the roughness decreased to 1.02 nm, indicating a smoother film surface. The phase image revealed a slight reduction in the nanoscale fibrillar structure of the active layer, potentially arising from the additive-promoted donor–acceptor miscibility, which partially optimized the phase separation structure. This uniform and smooth surface can optimize the contact with the interfacial layer, which is beneficial for charge transport. Figure 5c,d, respectively, present the 2D-GIWAXS patterns and the corresponding one-dimensional line-cut profiles in the OOP and IP directions for the blend films. The pristine blend film exhibited diffraction peaks at q = 1.70 Å^−1^ in the OOP direction and q = 0.291 Å^−1^ in the IP direction, corresponding to the (010) π-π stacking and (100) lamellar stacking signals, with calculated stacking distances of 3.70 Å and 21.59 Å, respectively. Upon the incorporation of the additive into the blend film, the OOP π-π stacking and IP lamellar stacking diffraction vectors increased to 1.71 Å^−1^ and 0.295 Å^−1^, and the corresponding packing distances are 3.68 Å^−1^ and 21.29 Å^−1^, indicating a reduction in the molecular stacking distances of the active layer components. The crystalline coherence length (CCL) in the IP (100) direction increased from 34.48 Å to 40.1 Å. The increased crystalline coherence length and the more tightly ordered molecular packing in the IBZN-treated film are anticipated to facilitate charge separation and carrier transport processes. The results of these calculations are summarized in Table S8.

**Figure 6:**
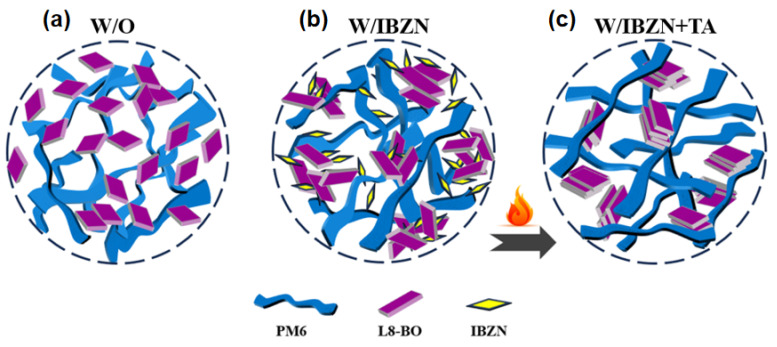
Schematic illustration of the active layer morphology (**a**) before and (**b**) after the incorporation of IBZN; (**c**) with IBZN after annealing treatment.

The favorable miscibility between IBZN and L8-BO likely results in a preferential distribution of IBZN molecules in the vicinity of L8-BO. The strong intermolecular interactions between IBZN and L8-BO modulate the acceptor’s aggregation, leading to enhanced crystallinity and a more favorable molecular orientation. Simultaneously, the presence of IBZN also improves the miscibility between the donor and acceptor. During the annealing process, the volatilization of IBZN facilitates the diffusion and migration of PM6 molecules into the resulting free space, enabling the formation of appropriate π-π stacking arrangements with L8-BO molecules. This process mitigates excessive L8-BO aggregation and promotes efficient charge transfer between PM6 and L8-BO. Consequently, the phase separation dimensions of the donor and acceptor are effectively controlled. This optimized phase separation, coupled with the smoother and more uniform film morphology, promotes efficient exciton dissociation and charge transport, as well as optimizing interfacial charge transfer characteristics, ultimately leading to a significant enhancement in device performance. The operational mechanism of the IBZN additive is schematically depicted in Figure 6.

To ascertain the versatility of IBZN in other NFA systems, device optimization was conducted based on PM6: Y6 and PM6: BTP-eC9 blends. The molecular structures of BTP-eC9 and Y6 are provided in Figure S12. The current density–voltage (*J*-*V*) characteristics are presented Figure S13, and the corresponding photovoltaic performance parameters are summarized in the Table S9. The control devices based on PM6: Y6 and PM6: BTP-eC9 exhibited PCEs of 15.9% and 16.51%, respectively. Following IBZN optimization, a significant improvement in both *J*_SC_ and FF was observed, resulting in enhanced PCEs of 17.23% and 18.12%, respectively. The EQE spectra for both sets of devices, illustrated in Figure S14, demonstrate a substantial increase in the EQE response across a broad spectral range for the devices incorporating IBZN. The discrepancy between the *J*_SC_ values derived from the EQE spectra and the *J*-*V* measurements remained within 4%. These findings suggest that IBZN modification can serve as an efficacious strategy for modulating the performance of PSC devices.

## 4. Conclusions

In summary, we introduced a structurally simple additive molecule, IBZN, with a large dipole moment into the active layer. Theoretical calculations, UV-Vis absorption spectroscopy, and morphological characterizations collectively demonstrate that the highly dipolar IBZN molecule can strongly interact with the acceptor component, significantly altering its molecular packing and crystallization behavior. Moreover, the incorporation of IBZN enhances the compatibility between the donor and acceptor materials, achieving precise control over phase separation size and optimizing the morphology of the active layer. As a result, the characteristics of charge separation and transport are significantly improved. Devices fabricated using the PM6:L8-BO system with IBZN exhibited an excellent fill factor (FF) of 79.54%, which increased the PCE from 17.49% to 18.77%. Additionally, IBZN demonstrated remarkable performance modulation effects in systems employing other Y-series acceptors. Specifically, the PCE of devices based on the PM6:Y6 and PM6:BTP-eC9 systems increased from 15.9% to 17.23% and from 16.51% to 18.12%, respectively. Our work validates the outstanding role of IBZN, a low-cost, structurally simple, and high-dipole moment volatile solid additive, in regulating the morphology of the active layer and optimizing device performance, providing insights for the further development of novel high-performance additives.

## Figures and Tables

**Table 1:** Photovoltaic performance parameters of the devices.

Conditions	*V*_OC_ (V)	*J*_SC_ (mA/cm^2^)	*J*_SC_ ^a^ (mA/cm^2^)	FF (%)	PCE ^b^ (%) Max.	PCE ^c^ (%) Avg.
PM6: L8-BO (w/o)	0.905	25.46	24.65	75.87	17.49	17.31 ± 0.18
PM6: L8-BO (w/IBZN)	0.898	26.28	25.27	79.54	18.77	18.65 ± 0.22

^a^ The integrated current density derived from EQE measurements. ^b^ The maximum power conversion efficiency (PCE) achieved. ^c^ The average PCE obtained from 10 independent devices.

**Table 2:** Optimization of IBZN concentration for PM6:L8-BO-based devices (annealing at 100 °C, 10 min).

Concentration of IBZN	*V*_OC_ (V)	*J*_SC_ (mA/cm^2^)	FF (%)	PCE (%)
40 wt%	0.901	25.69	77.3	17.91
70 wt%	0.898	26.28	79.54	18.77
100 wt%	0.894	26.04	79.09	18.42
150 wt%	0.889	25.96	78.55	18.14

**Table 3:** Optimization of annealing temperature for PM6:L8-BO-based devices (annealing for 10 min).

Annealing Temperature	*V*_OC_ (V)	*J*_SC_ (mA/cm^2^)	FF (%)	PCE (%)
90 °C	0.901	26.10	77.77	18.30
100 °C	0.898	26.28	79.54	18.77
110 °C	0.894	25.91	78.17	18.12

## Data Availability

The data are contained within the article and Supplementary Materials.

## Supplementary Materials

The following supporting information can be downloaded at https://www.mdpi.com/article/10.3390/polym17101386/s1: Figure S1: The Fourier transform infrared (FTIR) spectra of several thin film samples. Figure S2: The changes observed in the IBZN solution spin-coated on silicon substrates (a) before and (b) after the annealing process. Figure S3: The absorption coefficient spectra of the PM6: L8-BO blend film, both before and after the incorporation of IBZN. Figure S4: Top and lateral views of the DFT-optimized global minimum geometries (B3LYP-D3/LANL2DZ level) for a dimeric model of the PM6 polymer donor, as well as for the L8-BO molecular acceptor and the IBZN additive. Figure S5: Top views and Gibbs free energies of formation for the molecular complexes of PM6: IBZN and L8-BO: IBZN. Figure S6: J-V curves observed the optimization of IBZN concentration and annealing temperature for PM6: L8-BO-based devices. Figure S7: The chemical structures of the four halogenated benzonitrile derivatives—4-fluorobenzonitrile (FBZN), 4-chlorobenzonitrile (CBZN), 4-bromobenzonitrile (BBZN), and 4-iodobenzonitrile (IBZN). Figure S8: The *J-V* curves of the PM6:L8-BO-based devices using the four halogenated derivatives after optimization. Figure S9: The height maps, phase maps, and three-dimensional (3D) height maps (from left to right) of the surface morphology of (a) the pristine PM6 film and (b) PM6 films containing the additive IBZN. Figure S10: The height maps, phase maps, and three-dimensional (3D) height maps (from left to right) of the surface morphology of (a) the pristine L8-BO film and (b) L8-BO films containing the additive IBZN. Figure S11: The two-dimensional grazing-incidence wide-angle X-ray scattering (2D-GIWAXS) patterns of the pristine (a) L8-BO film and (b) L8-BO film with the incorporated IBZN, respectively. (c) The corresponding one-dimensional line cuts along the out-of-plane (OOP) and in-plane (IP) directions for both sets of films. Figure S12: The molecular structure of (a) BTP-ec9 and (b) Y6. Figure S13: *J-V* curves based on (a) PM6:Y6 and (b) PM6:BTP-ec9 with and without the optimization of IBZN. Figure S14: EQE curves based on (a) PM6: Y6 and (b) PM6: BTP-ec9 with and without the optimization of IBZN. Table S1: DFT-calculated Gibbs free energies (B3LYP-D3/LANL2DZ level) for the optimized structures of a dimer model of the PM6 polymer donor, the L8-BO acceptor, and the IBZN additive. Table S2: DFT-calculated Gibbs free energies of formation (ΔG_of_), intermolecular interaction energies (*E*_interaction_), intermolecular bonding energies (*E*_bonding_), and reorganization energies (*E*_reorganization_) for the different molecular complexes. Table S3: The measured water and diiodomethane contact angles, along with the calculated surface energy values, for the series of thin films. Table S4: Details of the Flory–Huggins interaction parameters derived from the thin film contact angle measurements. Table S5: The photovoltaic performance parameters of the PM6: L8-BO-based devices using the four halogenated derivatives after optimization. Table S6: The relevant parameters extracted from the exciton dissociation efficiency measurements (*J*_ph_-*V*_eff_ curve). Table S7: The relevant parameters extracted from *J*^1/2^-*V* curves obtained via the space-charge-limited current (SCLC) method. Table S8: The relevant parameters extracted from the GIWAXS analysis of the thin film series. Table S9: Performance parameters of devices based on the PM6:Y6 and PM6: BTP-ec9 systems. References [42,43,44,45,46] are cited in the Supplementary Materials.
