# Supplementary material for: 4-Iodobenzonitrile as Effective Solid Additive for High-Efficiency Polymer Solar Cells
Source: Polymers (Basel). 2025 May 18;17(10):1386. doi: 10.3390/polym17101386 (PMC12115044; doi:10.3390/polym17101386)
Supplement: Supplementary file 1 [file polymers-17-01386-s001.zip › polymers-3628706-supplementary.pdf]

# Sumplementary information

## 4-Iodobenzonitrile as Effective Solid Additive for High-efficiency Polymer Solar Cells

**Jiayu Li [1], Chuanchen Cai[1] , Yuechen Li [1,2], Changbiao Ma[1] , Sergio Gámez-Valenzuela[3] Yixiao Liu[1], Jianfeng Li[1*] , Xiaochen Wang[1*] and Yongfang Li[1,2]**

[1] School of Materials Science and Engineering, Shaanxi Normal University, Xi'an, Shaanxi710119, China
[2] Beijing National Laboratory for Molecular Sciences, CAS Key Laboratory of Organic Solids, Institute of Chemistry, Chinese Academy of Sciences, Beijing 100190, China
[3] Shenzhen Key Laboratory of Printed Electronics, Department of Materials Science and Engineering, Southern University of Science and Technology, Shenzhen, Guangdong 518055, China
* Correspondence: lijf0630@snnu.edu.cn; wangxc@snnu.edu.cn

## Experimental details

### *J-V* and EQE measurements

The current density–voltage (*J-V*) characteristics were measured using a Class AAA solar simulator (Beijing Zolix Instruments Co., Ltd.) and a Keithley 2400 source meter under standard solar illumination conditions (AM1.5G, 100 mW/cm$^2$). The light intensity of the solar simulator was calibrated using a standard silicon reference cell (Beijing Zolix Instruments Co., Ltd.). The EQE values were measured with an QE-R solar quantum efficiency test system (Enlitech Co., Ltd., Taiwan, China).

### UV-Vis absorption measurement

UV-Vis–NIR spectra were obtained on a Shimadzu UV3600 spectrophotometer.

### TGA measurement

Thermogravimetric analysis (TGA) was performed on a Q-600/Q1000 thermogravimetric analyzer at a heating rate of 10 °C/min under a nitrogen atmosphere.

### FTIR measurement

FTIR measurements were conducted using the Thermo Scientific™ Nicolet™ iS10 FTIR.

### AFM Characterization

Tapping mode was employed, and the height map, 3D height map, and phase image were all obtained using a Bruker Dimension Icon atomic force microscope. The root mean square roughness (RMS) values of the height images were obtained from the entire scan area (2 × 2 μm).

**Contact angle measurement**

Contact angles were measured using a KRUSS-DSA100 contact angle tester. Surface energy of the material can be divided into dispersive and polar components using the Owens–Wendt method:

$$\gamma = \gamma^{d} + \gamma^{p}$$

Based on the contact angle of the two different solvents, the dispersion surface energy and the polar surface energy can be calculated using the following formula.

$$(1 + \cos\theta)\gamma_{L} = 2\sqrt{\gamma_{S}^{d}\gamma_{L}^{d}} + 2\sqrt{\gamma_{S}^{P}\gamma_{L}^{P}}$$

θ is the contact angle of solvent, $\gamma_{L}$ is the surface energy of the solvent, $\gamma_{S}^{d}$ and $\gamma_{S}^{p}$ refer to the dispersive and polar surface energy of the solid, respectively; $\gamma_{L}^{d}$ and $\gamma_{L}^{P}$ refer to the dispersive and polar surface energy of the solvent, respectively. For water, the values of $\gamma_{L}^{d}$, $\gamma_{L}^{P}$, and $\gamma_{L}$ are 21.8, 51, and 72.8 mJ $m^{-2}$ . For diiodomethane, $\gamma_{L}$ is 50.8mJ $m^{-2}$. The Flory–Huggins interaction parameter can be calculated using the following equation:

$$\chi_{ab} = K\,(\sqrt{\gamma_{a}} - \sqrt{\gamma_{b}})^{2}$$

where $\gamma_a$ and $\gamma_b$ are the surface energy of material a and b, respectively.

**Computational Methods**

The molecular geometries for a dimeric model of PM6 polymer, L8-BO molecular acceptor and the IBZN additive were optimized at the density functional theory (DFT) level using the GAUSSIAN16 program. The hybrid generalized gradient approximation (GGA) functional B3LYP [1,2] was used together with the LANL2DZ basis set [3,4]. Note that these calculations include an explicit D3 correction for dispersion [5].

For molecular complexes, six plausible molecular arrangements were considered for each system. They consider the interaction of the IBZN additive with the different moieties of both electron donor and electron acceptor systems, in both parallel and antiparallel orientations. The frequencies were computed at the same level of theory as the geometry optimizations, aiming to obtain the Gibbs free energies at 298.15 K. No imaginary frequencies were observed. The Gibbs free energies of formation were calculated by the equation $\Delta G^{o}_{f} = G_{Com} - (G_{M1} + G_{M2})$, where $G_{Com}$ is the Gibbs free energy of the complex, and $G_{M1}$ and $G_{M2}$ are the Gibbs free energies of the interacting molecules. On the other hand,

bonding energies ($E_{bonding}$) were calculated as the difference between the total energies of the complexes and the optimized corresponding interacting molecules. Interaction energies ($E_{interaction}$) were calculated as the difference between the total energies of the complexes and the energies of the interacting molecules in the conformation that they adopt to form the complex. Finally, reorganization energies ($E_{reorganization}$) were calculated as the difference between $E_{bonding}$ and $E_{interaction}$.

## GIWAXS Characterization

GIWAXS measurements were conducted on a Xenocs-SAXS/WAXS system with an X-ray wavelength of 1.54189 Å, the X-ray tube power was 30 W. Pilatus3R 300 K was used as a 2D detector, the detector pixel size was 172 μm, and the sample-to-detector distance was 150 mm. Samples were prepared on Si/PEDOT: PSS substrates under the same conditions as those used for device fabrication.

## Charge carrier mobility measurement

Hole-only and electron-only devices for SCLC measurements were fabricated with similar methods in architectures of ITO/PEDOT:PSS/active layer/MoO3 and ITO/ZnO/active layer/PDINN/Ag, respectively. The ZnO layer was fabricated using a sol–gel method, and silver electrodes were deposited by thermal evaporation in vacuum. All device fabrications were performed in a nitrogen gas-filled glove box except for the deposition of PEDOT:PSS and ZnO layers.

The *J-V* curves measured on hole-only and electron-only devices were fitted to $J = 9\varepsilon_0\varepsilon_r\mu V^2/8L^3$, where $J$ is the current density; L is the film thickness of the active layer; μ is the hole or electron mobility; $\varepsilon_r$ is the relative dielectric constant (for organic semiconductor materials, a value of 3 is typically adopted); $\varepsilon_0$ is the permittivity of free space ($8.85\times10^{-12}$ F $m^{-1}$); $V$ is the internal voltage in the device; $V = V_{appl}-V_{bi}$, where $V_{appl}$ is the applied voltage to the device; $V_{bi}$ is the built-in voltage.

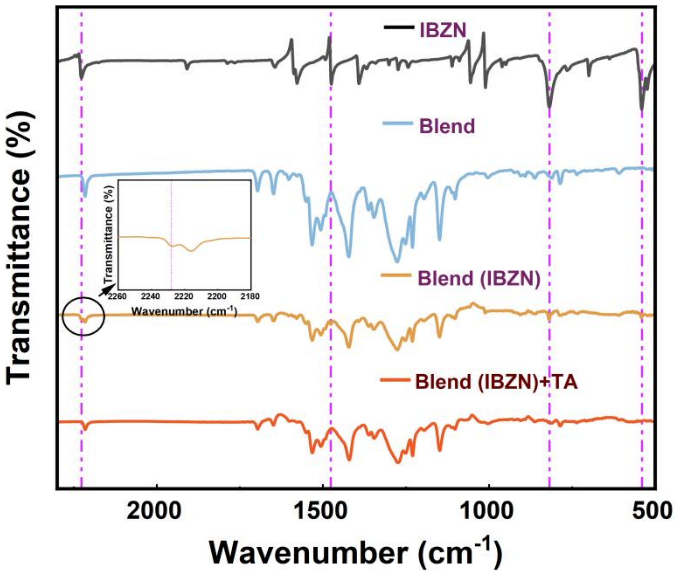


**Figure S1.** The Fourier transform infrared (FTIR) spectra of several thin film samples.

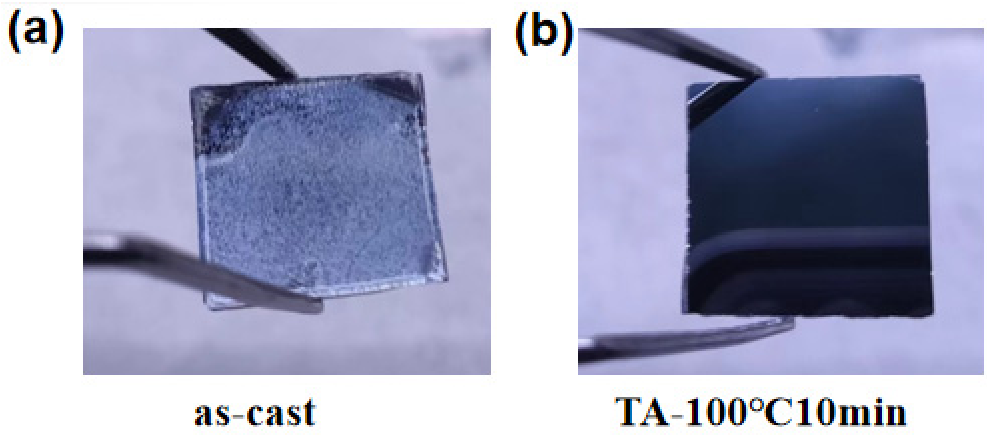


**Figure S2.** The changes observed in the IBZN solution spin-coated on silicon substrates（a）before and（b）after the annealing process.

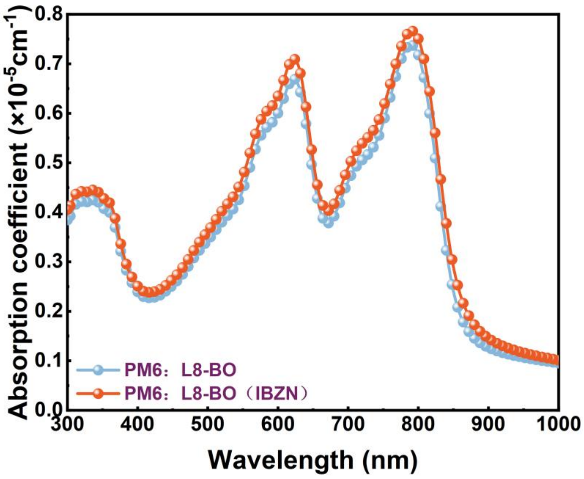


**Figure S3.** The absorption coefficient spectra of the PM6:L8-BO blend film, both before and after the incorporation of IBZN.

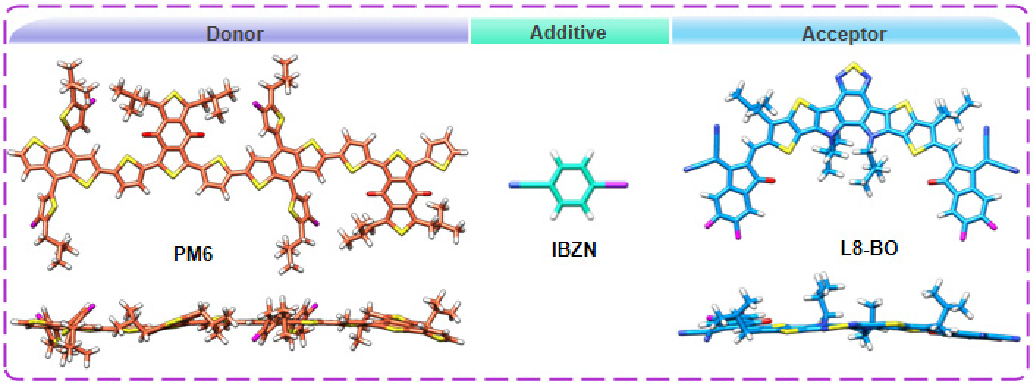


**Figure S4.** Top and lateral views of the DFT-optimized global minimum geometries (B3LYP-D3/LANL2DZ level) for a dimeric model of the PM6 polymer donor, as well as for the L8-BO molecular acceptor and the IBZN additive.

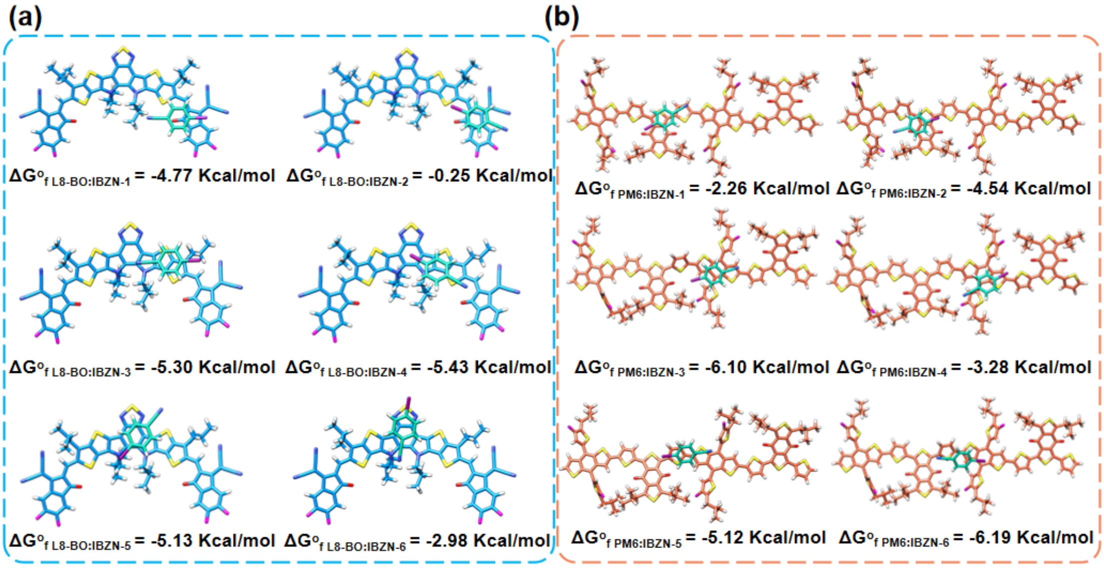


**Figure S5.** Top views and Gibbs free energies of formation for the molecular complexes of PM6:IBZN and L8-BO:IBZN.

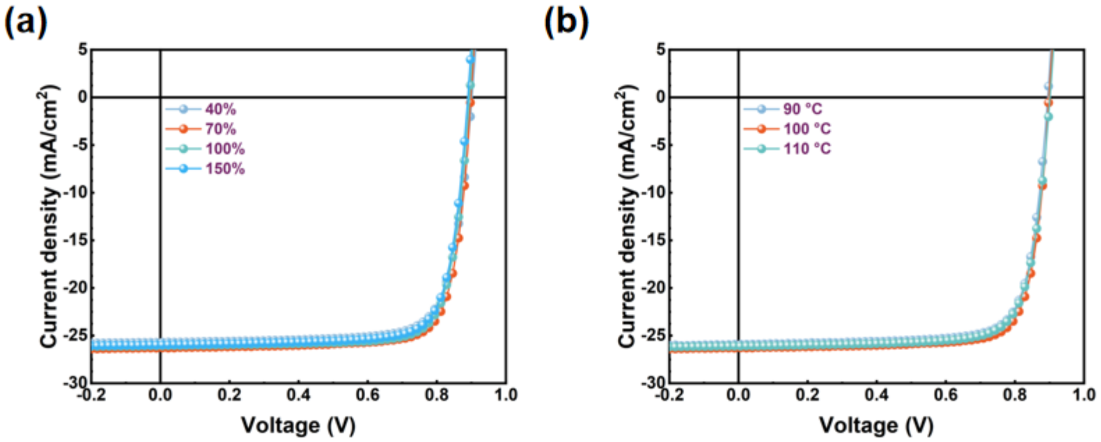


**Figure S6.** J-V curves observed the optimization of (a) IBZN concentration and (b) annealing temperature for PM6:L8-BO-based devices.

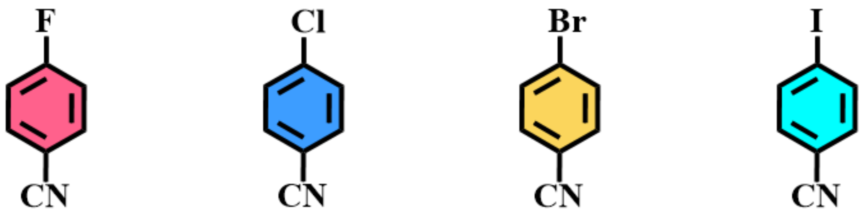


**Figure S7.** The chemical structures of the four halogenated benzonitrile derivatives—4-fluorobenzonitrile (FBZN), 4-chlorobenzonitrile (CBZN), 4-bromobenzonitrile (BBZN), and 4-iodobenzonitrile (IBZN).

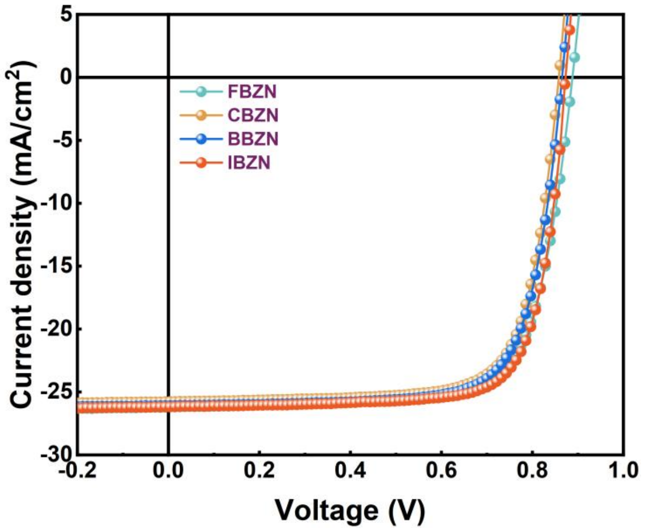


**Figure S8.** The *J-V* curves of the PM6:L8-BO-based devices using the four halogenated derivatives after optimization.

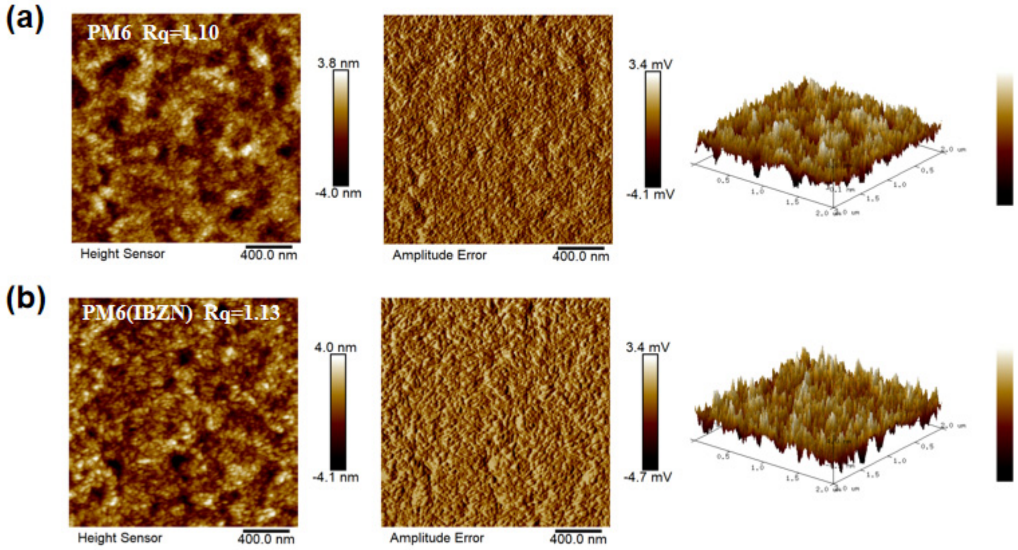


**Figure S9.** The height maps, phase maps, and three-dimensional (3D) height maps (from left to right) of the surface morphology of (a) the pristine PM6 film and (b) PM6 films containing the additive IBZN.

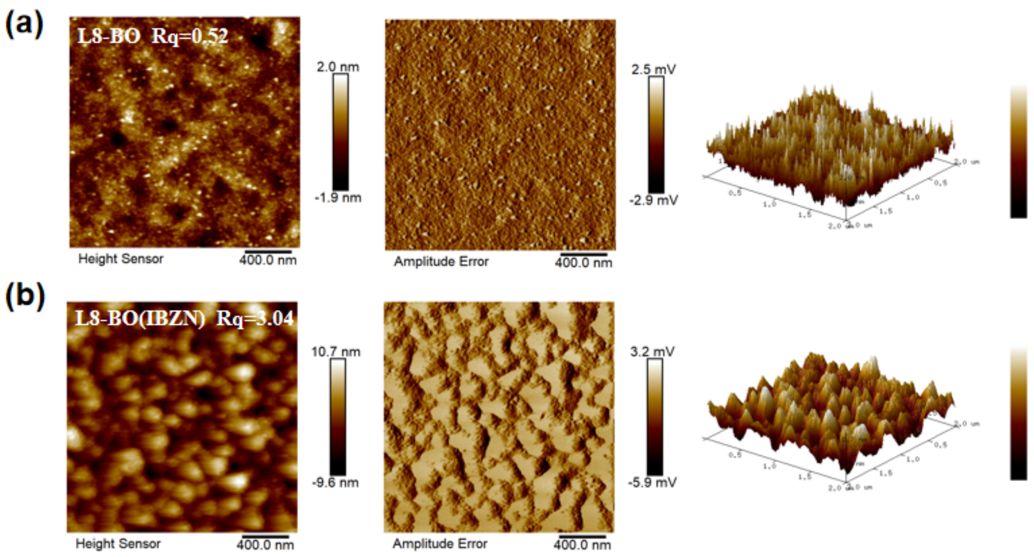


**Figure S10.** The height maps, phase maps, and three-dimensional (3D) height maps (from left to right) of the surface morphology of (a) the pristine L8-BO film and (b) L8-BO films containing the additive IBZN.

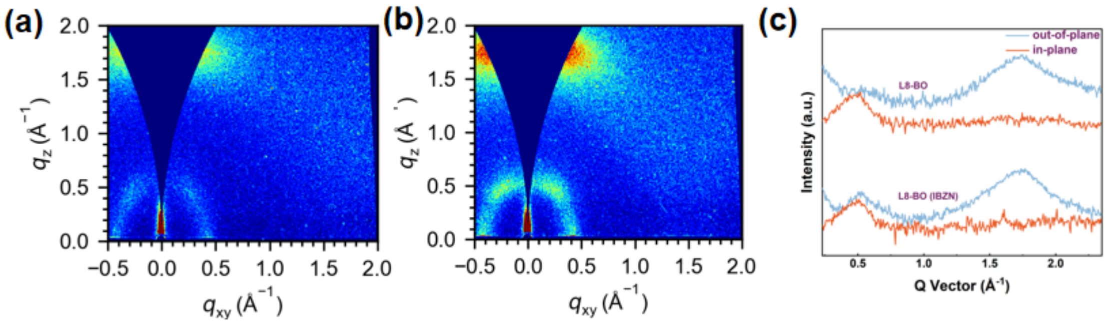


**Figure S11.** The two-dimensional grazing-incidence wide-angle X-ray scattering (2D-GIWAXS) patterns of the pristine (a) L8-BO film and (b) L8-BO film with the incorporated IBZN, respectively. (c) The corresponding one-dimensional line cuts along the out-of-plane (OOP) and in-plane (IP) directions for both sets of films.

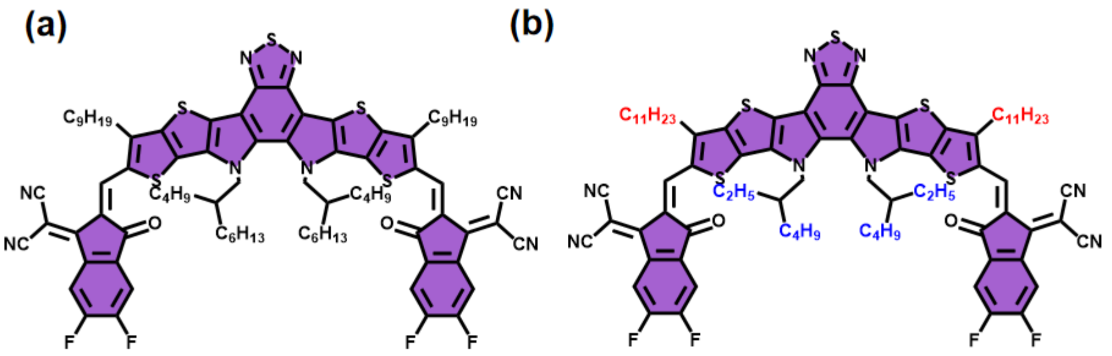


**Figure S12.** The molecular structure of (a) BTP-ec9 and (b) Y6.

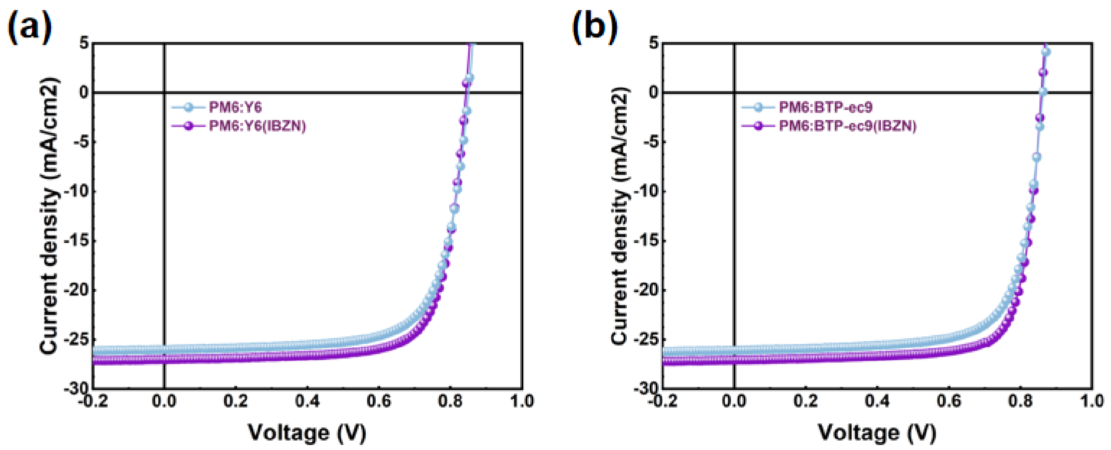


**Figure S13.** *J-V* curves based on (a) PM6:Y6 and (b) PM6:BTP-ec9 with and without the optimization of IBZN.

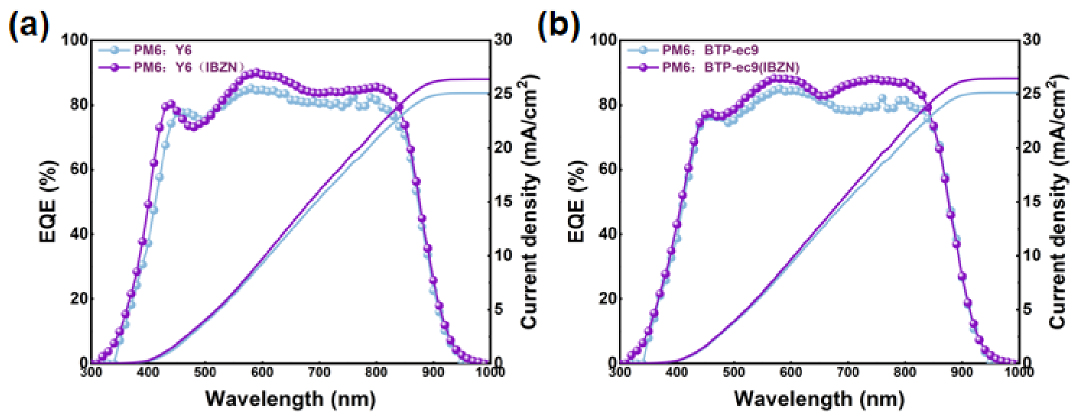


**Figure S14.** EQE curves based on (a) PM6:Y6 and (b) PM6:BTP-ec9 with and without the optimization of IBZN.

**Table S1**. DFT-calculated Gibbs free energies (B3LYP-D3/LANL2DZ level) for the optimized structures of a dimer model of the PM6 polymer donor, the L8-BO acceptor, and the IBZN additive.

| Sample | Optimal energy (HF) | ΔGo (HF) |
|---|---|---|
| PM6 | -4878.63192892 | -4877.147859 |
| L8-BO | -3349.52366448 | -3348.743766 |
| IBZN | -335.21798698 | -335.163252 |

**Table S2.** DFT-calculated Gibbs free energies of formation ($\Delta G^o_f$), intermolecular interaction energies ($E_{interaction}$), intermolecular bonding energies ($E_{bonding}$), and reorganization energies ($E_{reorganization}$) for the different molecular complexes.

| Sample | Energy (HF) | ΔGo (HF) | Energy (HF) | Energy (HF) | $\Delta G^o_f$ (Kcal/mol) | $E_{bonding}$ (Kcal/mol) | $E_{interaction}$ (Kcal/mol) | $E_{reorganization}$ (Kcal/mol) |
|---|---|---|---|---|---|---|---|---|
| **L8-BO:IBCN-1** | -3684.7725572 | -3683.914625 | -3349.5229383 | -335.2178499 | -4.77 | -19.39 | -19.94 | 0.54 |
| **L8-BO:IBCN-2** | -3684.7619737 | -3683.907409 | -3349.5233850 | -335.2179560 | -0.25 | -12.75 | -12.95 | 0.19 |
| **L8-BO:IBCN-3** | -3684.7717609 | -3683.915460 | -3349.5224870 | -335.2178937 | -5.30 | -18.89 | -19.69 | 0.80 |
| **L8-BO:IBCN-4** | -3684.7707313 | -3683.915665 | -3349.5223720 | -335.2179200 | -5.43 | -18.24 | -19.10 | 0.85 |
| **L8-BO:IBCN-5** | -3684.7701747 | -3683.915192 | -3349.5224130 | -335.2179439 | -5.13 | -17.90 | -18.71 | 0.81 |
| **L8-BO:IBCN-6** | -3684.7692682 | -3683.911773 | -3349.5224014 | -335.2177960 | -2.98 | -17.33 | -18.24 | 0.91 |
| **PM6:IBCN-1** | -5213.8757315 | -5212.314705 | -4878.6303597 | -335.2177725 | -2.26 | -16.20 | -17.32 | 1.12 |
| **PM6:IBCN-2** | -5213.8828639 | -5212.318338 | -4878.6290282 | -335.2177677 | -4.53 | -20.68 | -22.63 | 1.96 |
| **PM6:IBCN-3** | -5213.8853151 | -5212.320827 | -4878.6301457 | -335.2177182 | -6.10 | -22.21 | -23.50 | 1.29 |
| **PM6:IBCN-4** | -5213.8797605 | -5212.316335 | -4878.6288631 | -335.2176762 | -3.28 | -18.73 | -20.85 | 2.12 |

| | | | | | | | | |
|---|---|---|---|---|---|---|---|---|
| **PM6:IBCN-5** | -5213.8806271 | -5212.319276 | -4878.6313972 | -335.2177509 | -5.12 | -19.27 | -19.75 | 0.48 |
| **PM6:IBCN-6** | -5213.8801781 | -5212.320971 | -4878.6295776 | -335.2177271 | -6.19 | -18.99 | -20.63 | 1.64 |

**Table S3.** The measured water and diiodomethane contact angles, along with the calculated surface energy values, for the series of thin films.

| **Additive** | **Film** | **Contact angle (°)** | | **Surface energy,** $\gamma$ **（mN m**$^{-1}$**）** |
|---|---|---|---|---|
| | | **$H_2O$** | **$CH_2I_2$** | |
| W/O | PM6 | 102.7 | 48.8 | 34.98 |
| | L8-BO | 93.4 | 41.3 | 39.7 |
| W/IBZN | PM6 | 105.6 | 52.7 | 32.7 |
| | L8-BO | 99.0 | 46.9 | 35.95 |
| | IBZN | 87.4 | 36.3 | 42.8 |

**Table S4.** Details of the Flory–Huggins interaction parameters derived from the thin film contact angle measurements.

| | **D and A(w/o)** | **D and A(IBZN)** | **D and IBZN** | **A and IBZN** |
|---|---|---|---|---|
| $\chi$ (K) | 0.149 | 0.076 | 0.394 | 0.058 |

**Table S5.** The photovoltaic performance parameters of the PM6:L8-BO-based devices using the four halogenated derivatives after optimization.

| **Conditions（PM6：L8-BO）** | **V*oc* (V)** | **J*sc* (mA/cm2)** | **FF（%）** | **PCE（%）** |
|---|---|---|---|---|
| W/FBZN | 0.888 | 26.18 | 73.91 | 17.18 |
| W/CBZN | 0.886 | 25.89 | 77.98 | 17.89 |
| W/BBZN | 0.892 | 26.17 | 77.85 | 18.17 |
| W/IBZN | 0.898 | 26.28 | 79.54 | 18.77 |

**Table S6.** The relevant parameters extracted from the exciton dissociation efficiency measurements ($J_{ph}$-$V_{eff}$ curve).

| **PM6：L8-BO** | **$J_{SC}$(mA cm$^{-2}$)** | **$J_{max}$(mA cm$^{-2}$)** | **$J_{sat}$(mA cm$^{-2}$)** | **$\eta_{diss}$(%)** | **$\eta_{coll}$(%)** |
|---|---|---|---|---|---|
| w/o | 25.28 | 21.88 | 26.34 | 95.9 | 83.1 |
| w/ibzn | 26.26 | 23.89 | 27.02 | 97.2 | 88.5 |

**Table S7.** The relevant parameters extracted from $J^{1/2}$-$V$ curves obtained via the space-charge-limited current (SCLC) method.

| **PM6：L8-BO** | **$\mu_h$(cm$^2$V$^{-1}$S$^{-1}$)** | **$\mu_e$(cm$^2$V$^{-1}$S$^{-1}$)** | **$\mu_h/\mu_e$** |
|---|---|---|---|
| w/o | $5.15\times10^{-4}$ | $2.76\times10^{-4}$ | 1.86 |
| w/ibzn | $7.23\times10^{-4}$ | $5.46\times10^{-4}$ | 1.32 |

**Table S8.** The relevant parameters extracted from the GIWAXS analysis of the thin film series.

| **Sample** | **Direction** | **Location(Å-1)** | **D-sapcing(Å)** | **FWHM(Å$^{-1}$)[a]** | **CCL(Å)[b]** |
|---|---|---|---|---|---|
| PM6:L8-BO | OOP | 1.70 | 3.70 | 0.252 | 22.44 |
| | IP | 0.291 | 21.59 | 0.164 | 34.48 |
| PM6:L8-BO(IBZN) | OOP | 1.71 | 3.68 | 0.259 | 21.83 |
| | IP | 0.295 | 21.29 | 0.141 | 40.1 |
| L8-BO | OOP | 1.74 | 3.61 | 0.267 | 21.18 |
| | IP | 0.480 | 13.08 | 0.164 | 34.48 |
| L8-BO(IBZN) | OOP | 1.75 | 3.59 | 0.211 | 26.8 |
| | IP | 0.498 | 12.61 | 0.147 | 38.47 |

[a] D-spacing= $2\pi/q$.

[b]CCL = $1.8\pi$/FWHM.

**Table S9.** Performance parameters of devices based on the PM6:Y6 and PM6:BTP-ec9 systems.

| **Blends** | **Conditions** | **$V_{OC}$ (V)** | **$J_{SC}$ (mA/cm$^2$)** | **FF（%）** | **PCE（%）** |
|---|---|---|---|---|---|
| PM6：Y6 | w/o | 0.850 | 25.99 | 71.96 | 15.90 |
| | w/IBZN | 0.843 | 27.33 | 74.74 | 17.23 |
| PM6：BTP-ec9 | w/o | 0.862 | 26.12 | 73.35 | 16.51 |
| | w/IBZN | 0.859 | 27.23 | 77.49 | 18.12 |

## References


1. Lee, C.; Yang, W.; Parr, R.G. Development of the Colle-Salvetti correlation-energy formula into a functional of the electron density. *Phys. Rev. B.* **1988**, *37*, 785.
2. Becke, A.D. Density-functional thermochemistry. III. The role of exact exchange. *J. Chem. Phys.* **1993**, *98*, 5648-5652.
3. Hay, P.J.; Wadt, W.R. Ab initio effective core potentials for molecular calculations. Potentials for K to Au including the outermost core orbitals. *J. Chem. Phys.* **1985**, *82*, 299-310.
4. Wadt, W.R.; Hay, P.J. Ab initio effective core potentials for molecular calculations. Potentials for main group elements Na to Bi. *J. Chem. Phys.* **1985**, *82*, 284-298.
5. Grimme, S.; Antony, J.; Ehrlich, S.; Krieg, H. A consistent and accurate ab initio parametrization of density functional dispersion correction (DFT-D) for the 94 elements H-Pu. *J. Chem. Phys.* **2010**, *132*.
